# Comparison of unplanned intensive care unit readmission scores: a prospective cohort study

**DOI:** 10.1186/2197-425X-3-S1-A472

**Published:** 2015-10-01

**Authors:** C Roehrig, RG Rosa, AM Ascoli, L Madeira, W Rutzen, J Maccari, P Balzano, AC Antonio, P Castro, RPd Oliveira, C Teixeira

**Affiliations:** Moinhos de Vento Hospital, Porto Alegre, Brazil

## Introduction

Early discharge from intensive care unit (ICU) may constitute a strategy of resource consumption optimization; however, unplanned readmission of hospitalized patients to an ICU is associated with a worse outcome.

## Objectives

To compare the effectiveness of the stability and workload index for transfer score (SWIFT), the sequential organ failure assessment score (SOFA) and simplified therapeutic intervention scoring system (TISS-28) in predicting unplanned ICU readmission or unexpected death in the first 48 hours after discharge from the ICU.

## Methods

We conducted a prospective cohort study in a single tertiary hospital in Southern Brazil. All adult patients admitted to the ICU for more than 24 hours from January 2008 to December 2009 were evaluated. SWIFT, SOFA and TISS-28 scores were calculated on the day of discharge from ICU. A stepwise logistic regression was conducted for evaluation of the effectiveness of these scores in predicting unplanned ICU readmission or unexpected death in the first 48 hours after discharge from the ICU. Moreover, we conducted a direct accuracy comparison among SWIFT, SOFA and TISS-28 scores.

## Results

In total 1277 patients were discharged from the ICU during the study period. The rate of unplanned ICU readmission or unexpected death in the first 48 hours after discharge from the ICU was 15% (192 patients). In the multivariate analysis,age (*P* = 0.001), length of ICU stay (*P* = 0.01), cirrhosis (*P* = 0.03), SWIFT (*P* = 0.001), SOFA (*P* = 0.01) and TISS-28 (*P* < 0.001) constituted predictors of unplanned ICU readmission or unexpected death. SWIFT, SOFA and TISS-28 scores showed similar predictive accuracy (AUC 0.65, 0.65 and 0.67, respectively, *P* = 0.58).

## Conclusions

SWIFT, SOFA and TISS-28 on the day of discharge from ICU may constitute important tools to predict ICU readmission or death. The present study did not find any accuracy difference among the three scores.
